# Correlation of patient-reported numbness around surgical scars with patient-reported outcome measures and joint awareness after knee replacement: a cohort study

**DOI:** 10.1186/s12891-021-04971-6

**Published:** 2022-01-03

**Authors:** Masafumi Itoh, Junya Itou, Umito Kuwashima, Ken Okazaki

**Affiliations:** Department of Orthopaedic surgery, Tokyo Women’s Medical University, 8–1, Kawada-Cho, Shinjuku-ku, Tokyo, 162-8666 Japan

**Keywords:** Knee replacement, Numbness, Joint awareness, Forgotten joint Score-12, Kneeling

## Abstract

**Background:**

Knee replacement is a very effective and indispensable treatment option for end-stage knee arthritis, and the number of cases has been increasing worldwide. A replaced knee joint without patient joint awareness is thought to be the ultimate goal of artificial knees. Joint awareness reportedly correlates with patient satisfaction. Although numbness around a replaced knee is a minor but common problem, its effect on postoperative outcome is controversial. Joint awareness also is sensitive to subtle abnormalities of the joint, so it must be negatively affected by numbness. Although numbness is minor, it cannot be ignored to further improve knee replacement outcomes. This study investigated the relationship between patient-reported numbness and other patient-reported outcome measures (PROMs), including joint awareness, and kneeling. We developed a numbness score based on a 5-point Likert scale on frequency of numbness, with an intraclass correlation coefficient of 0.76 and higher scores indicating less numbness.

**Methods:**

The numbness score, New Knee Society Score (KSS), Knee Injury and Osteoarthritis Outcome Score (KOOS), Forgotten Joint Score-12 (FJS-12), and other clinical and radiological data from 311 patients (394 primary knee replacements) were analyzed. Kneeling ability was evaluated by using kneeling-specific items in the KSS (KSS-Kneeling).

**Results:**

No numbness was found in 170 knees (43.1%), and some degree of numbness was found in the remaining 224 knees (56.9%). The numbness score showed weak-to-moderate correlations with KSS-Symptoms (*r* = 0.44), KSS-Satisfaction (*r* = 0.41), KSS-Activities (*r* = 0.29), and all KOOS subscales (*r* = 0.23–0.44), and FJS-12 (*r* = 0.42). Multiple regression analyses suggested that midline incision positively affected the numbness score over the anteromedial incision (*p* = 0.04) and that a better numbness score (*p* = 0.001), male sex (*p* < 0.0001), and better postoperative knee flexion angle (0.04) positively affected kneeling.

**Conclusions:**

The numbness score positively correlated with PROMs and positively affected kneeling. Knee replacements performed via an anteromedial incision may be at higher risk for numbness.

**Supplementary Information:**

The online version contains supplementary material available at 10.1186/s12891-021-04971-6.

## Background

Knee arthroplasty is an effective treatment option for osteoarthritis (OA), osteonecrosis (ON), and rheumatoid arthritis (RA) of the knee [1–6]. Especially in the end-stages of these conditions when there is no alternative treatment available, knee arthroplasty is a very effective and indispensable technique [6]. In 2010, knee replacement in the United States was performed in 1.5% of the general population and 10.4% of the population > 80 years old [7], and the number of cases is expected to increase worldwide [8, 9]. Major complications, such as deep infection, loosening, and instability, often require revision surgery, whereas minor complications, such as discomfort around the scar [10] and noise from the knee [11], seldom require revision. In addition, minor complications related to wounds are relatively common [12], and wound-related complications have potential risks of developing into major complications that require further surgery. Other researchers have reported that 46–98% of patients who underwent knee replacement complained of numbness around the knee postoperatively [10, 13, 14]. Jariwala et al. [13] reported that 53% of 258 total knee arthroplasties (TKAs) resulted in complaints of numbness around the operated knee, but the numbness did not correlate with patient-reported outcome measures (PROMs), such as the New Knee Society Score (KSS) [15] at 1 year after the surgery. Blackburn et al. [14] reported that 68% of 56 TKAs resulted in complaints of numbness but the numbness did not correlate with the Western Ontario and McMaster Universities Osteoarthritis Index (WOMAC) score [16] or the Knee Injury and Osteoarthritis Outcome Score (KOOS) [17]. However, Blackburn et al. also found that numbness around the operated knee correlated with kneeling difficulty and that kneeling difficulty correlated with poor results of WOMAC and KOOS. In fact, it was reported that 56–82% of patients who underwent knee arthroplasty complained of kneeling difficulty [11, 18, 19]. Therefore, although numbness around the replaced knee is a relatively common minor complication, how it may be associated with clinical outcomes remains controversial.

It is assumed that numbness is related to discomfort rather than pain that most conventional PROMs aim to assess. In 2012, Behrend et al. developed the Forgotten Joint Score-12 (FJS-12) [20] as a PROM that evaluates joint awareness, detects subtle discomfort, has a low ceiling effect, and was found to be useful in evaluating joint replacements. Subsequently, many authors have used the FJS-12 to report the outcomes of knee replacement [21–25]. Although some authors found that numbness after knee replacement did not correlate with conventional PROMs [11, 13, 14], the FJS-12, which assesses joint awareness, may be able to detect the effect of numbness. To further improve knee replacement outcomes, although numbness is minor, it cannot be ignored.

This study aimed to investigate the correlation between numbness around the surgical scar after knee replacement and PROMs, including the FJS-12, and to assess the influence of this numbness on kneeling in a larger cohort than in previous studies [10, 11, 13, 14, 26]. We hypothesized that worse numbness would be correlated with poorer postoperative PROMs and would adversely affect kneeling.

## Methods

### Patients and data collection

This retrospective cohort study was approved by the institutional ethics committee of Tokyo Women’s Medical University, Tokyo, Japan (approval number 4578) and performed in accordance with the ethical standards of the Declaration of Helsinki. We retrospectively investigated 514 knee replacements in 404 patients performed at our institution between May 2007 and January 2019. All procedures were performed by any of five specialist knee surgeons who were trained in both knee replacement and sports medicine.

**Fig. 1:**
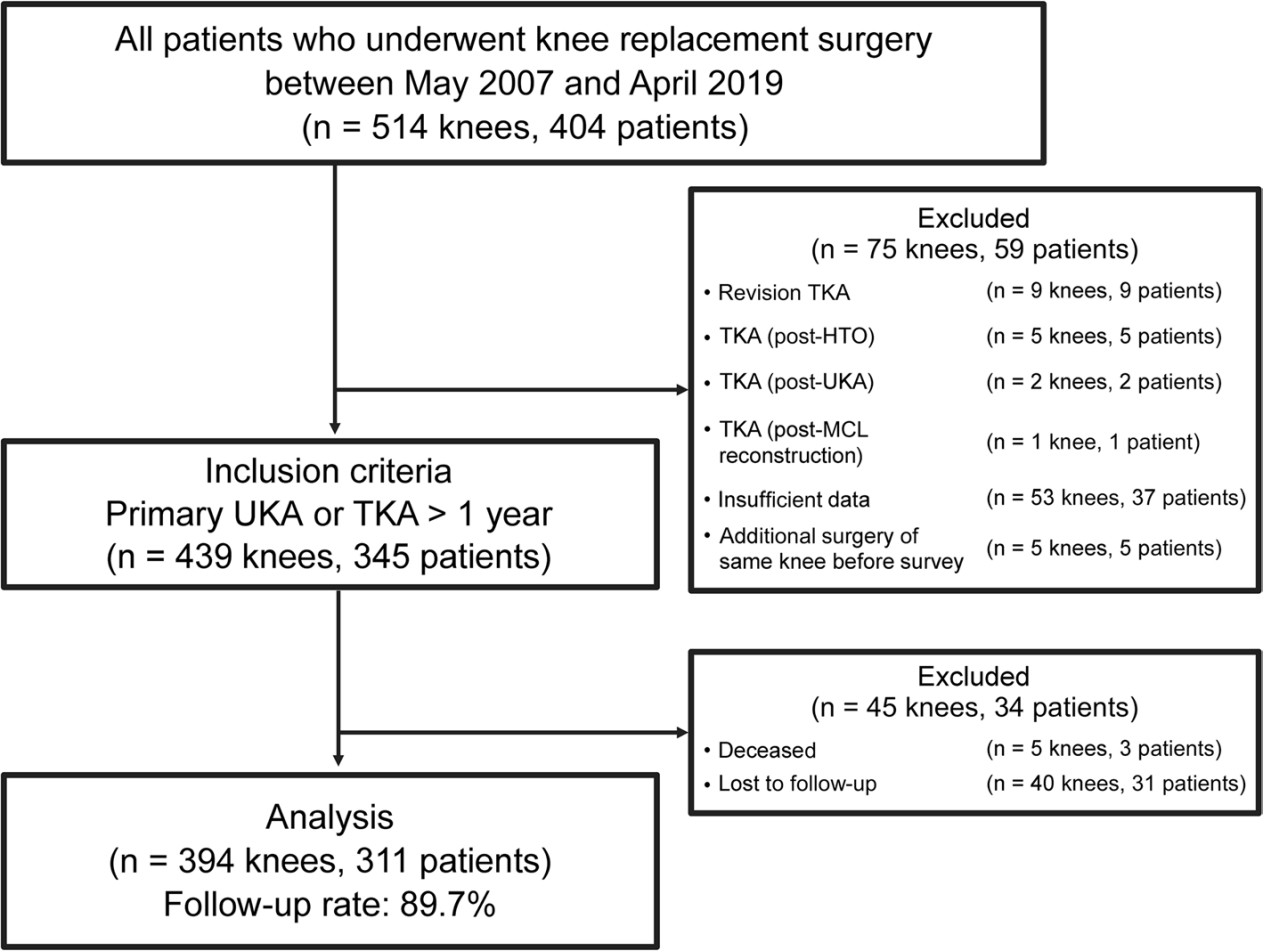
Flowchart for this retrospective study. The flowchart shows the cohort of patients who underwent knee replacement surgery at our institution, surgical details, mortality, and loss to follow-up. HTO, high tibial osteotomy; MCL, medial collateral ligament; TKA, total knee arthroplasty; UKA, unicondylar knee arthroplasty

Unicondylar knee arthroplasty (UKA) was indicated for patients diagnosed with medial OA or medial ON of the knee with varus malalignment of < 20° and flexion contracture of < 20° in which the anterior and posterior cruciate ligaments were functional, and the lateral and patellofemoral compartments were healthy or asymptomatic with very mild changes. TKA was indicated for patients diagnosed with other than knee infection without limitation of the malalignment degree, movement restriction range, impaired compartments number, and cruciate ligament residual function. The postoperative alignment of TKA was aimed for mechanical alignment, where the hip, knee, and ankle centers were aligned in a straight line in the coronal plane [27] using conventional instruments without computer navigation or robotic assisted surgery. The inclusion criteria were patients who underwent primary UKA or TKA without restrictions on body mass index (BMI), age, worker compensation status, or psychiatric disorders such as anxiety and depression. The exclusion criteria were revision knee arthroplasty, past major knee surgery (such as ligament reconstruction or knee osteotomy on the same knee), any additional surgery on the same knee, and incomplete records. Five knees had undergone additional surgery before the survey: three patients with three TKAs underwent irrigation and insert exchange due to infection, one patient following a UKA underwent open reduction of a dislocated mobile bearing insert, and one patient following a UKA underwent an arthroscopically assisted hemostatic procedure for hemarthrosis. Consequently, 439 knees in 345 patients met the eligibility criteria. After exclusion of fatal cases and patients who were lost to follow-up, 394 knees in 311 patients were enrolled in the study (follow-up rate, 89.7%; Fig. 1).

A post-hoc power analysis was calculated by using G*power (version 3.1.9.3, Institut für Experimentelle Psychologie, Heinrich Heine Universität, Dusseldorf, Germany). An effect size of 0.42 (see the result section) was calculated on the basis of Spearman’s correlation coefficients between the FJS-12 and numbness score. A power greater than 0.99 was calculated by using the effect size, sample size of 394 knees, and alpha level of 0.01.

Demographic and clinical data, including sex, age at surgery, age at survey completion, follow-up (months), BMI, diagnosis, preoperative Kellgren–Lawrence (K-L) [28] grading, surgical method used (UKA or TKA), type of skin incision (midline or anteromedial), type of arthrotomy (medial parapatellar, midvastus, trivector, or subvastus), and preoperative and postoperative extension and flexion angles of the knee were collected from the medical records and radiographs. Postoperatively, written informed consent and the KSS, KOOS, FJS-12, and numbness score (as described below) were obtained for each patient. The questionnaire item on kneeling in the KSS was defined as “KSS-Kneeling” and was used to assess kneeling ability. If a patient had undergone bilateral knee arthroplasty, the data for each knee were collected separately. Postoperative data, including PROMs, were collected at a minimum of 1 year postoperatively.

Table 1 shows the clinical and demographic data of the 394 knees in 311 patients. In total, 339 knees were diagnosed as OA and graded by using the K-L grading system. All UKAs were mobile bearing UKA (Oxford unicompartmental knee prosthesis. Zimmer Biomet Ltd., Bridgend, UK) via an anteromedial incision and midvastus approach. A midline or anteromedial incision and one of the arthrotomy approaches (medial parapatellar, trivector, midvastus, or subvastus) were selected for the TKA at the discretion of the surgeon.

**Table 1:** Demographics of 394 knees (311 patients)

Men: % (number of knees)	20.0% (79 knees)
Age at surgery (years)	72.9 ± 8.2
Age at survey completion (years)	75.2 ± 8.2
Follow-up (months)	28.0 ± 25.2
BMI at survey completion (kg/m^2^)	25.5 ± 4.3
Diagnosis: % (number of knees)	OA: 86.0% (339) ON: 7.4% (29) RA: 6.6% (26)
Preoperative K-L grade of 339 knees diagnosed OA: % (number of knees)	Grade 1: 0.3% (1) Grade 2: 5.6% (19) Grade 3: 18.9% (64) Grade 4: 75.2% (255)
Surgical method: % (number of knees)	TKA: 81.2% (320) UKA: 18.8% (74)
Skin incision: % (number of knees)	Midline: 51.5% (203) Anteromedial: 48.5% (191)
Arthrotomy: % (number of knees)	Medial parapatellar: 25.4% (100) Trivector: 3.5% (14) Midvastus: 22.6% (89) Subvastus: 48.5% (191)

*BMI* Body mass index, *K–L* Kellgren–Lawrence, *OA* Osteoarthritis, *ON* Osteonecrosis, *RA* Rheumatoid arthritis, *TKA* Total knee arthroplasty, *UKA* Unicondylar knee arthroplasty

### Numbness score

Given the subjective nature of numbness, the numbness score for this study was devised using a Likert scale-type questionnaire that had the same question format as that of the FJS-12 questionnaire. Patients were asked “Are you aware of numbness that bothers you around the surgical scar of your operated knee?” and were then asked to choose a reply from one of the following: “never,” “almost never,” “seldom,” “sometimes,” or “mostly,” which were assigned 4, 3, 2, 1, and 0 points, respectively. This meant that higher is the numbness score, less is the numbness. To calculate intraclass correlation (ICC) as a measure of numbness score reliability, the numbness scores were collected twice at intervals of ≤1 month from 40 randomly selected individuals included in the analysis.

### Statistical analysis

JMP Pro version 13.2.1 (SAS Institute Inc., Cary, NC, USA) was used. A *p*-value of < 0.05 was considered to be indicative of statistical significance. The Wilcoxon signed-rank test was used to evaluate differences between the pre- and postoperative numerical data. The differences in means between two groups were evaluated by performing the Mann–Whitney *U-*test. Spearman’s correlation coefficient was used to analyze correlations between the numbness score and each PROM. Table 2 shows the strength of the correlation coefficient (*r*) [29]. In addition, univariable and multivariable regression analyses with the numbness score and KSS-Kneeling as the response variables were performed to correct the confounding bias. The ICC (2,1) of the numbness score was calculated [30]. The test–retest reliability of the numbness score was confirmed, with an ICC (2,1) of 0.76.

**Table 2:** Definition of the strength of the correlation coefficients

Range of correlation coefficients (r)	Strength of the correlation
0 ≤ r < 0.1	Negligible
0.1 ≤ r < 0.4	Weak
0.4 ≤ r < 0.7	Moderate
0.7 ≤ r < 0.9	Strong
0.7 ≤ r < 1.0	Very strong

## Results

Preoperative flexion contracture was significantly greater in knees scheduled for TKA than for UKA (*p* < 0.0001). Postoperative flexion contracture was comparable between both groups (*p* = 0.32). Preoperative flexion was significantly less in knees scheduled for TKA than UKA (*p* < 0.0001). Postoperative flexion was significantly less in TKAs than UKAs (*p* < 0.0001). Postoperatively, both groups significantly improved in both extension and flexion (*p* < 0.0001) (Table 3).

**Table 3:** Comparison of preoperative and postoperative knee extension and flexion angle by surgical method

Type of surgery	TKA: 320 knees	UKA: 74 knees	*p*-value
Knee extension angle (°)			
Preoperative	8.7 ± 8.3	3.6 ± 4.6	**< 0.0001**
Postoperative	0.7 ± 2.3	0.3 ± 1.5	0.32
*p*-value	**< 0.0001**	**< 0.0001**	
Knee flexion angle (°)			
Preoperative	123.5 ± 15.7	132.8 ± 11.0	**< 0.0001**
Postoperative	132.8 ± 1.0	137.0 ± 8.0	**< 0.0001**
*p*-value	**< 0.0001**	**< 0.0001**	

The knee extension angle is expressed as a negative value for hyperextension and a positive value for limitation of extension.

Boldface means statistically significant.

*TKA* Total knee arthroplasty, *UKA* Unicondylar knee arthroplasty

**Fig. 2:**
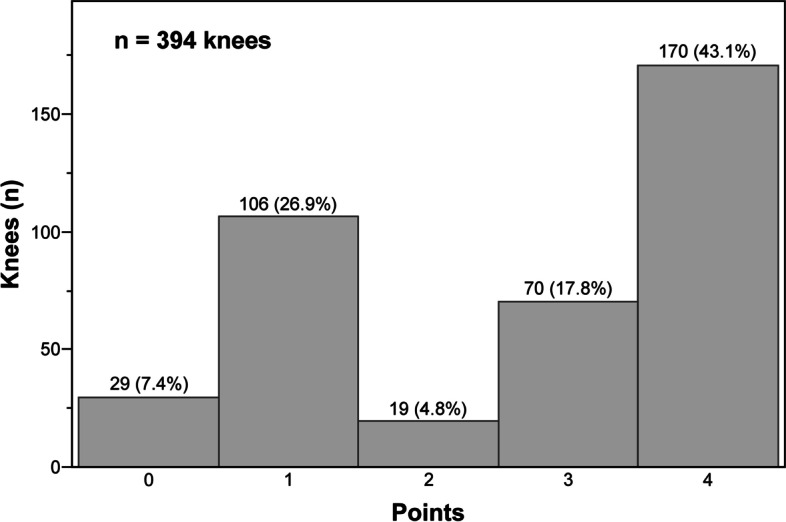
Histogram showing the numbness scores for 394 knees. A score of 4 points indicates no numbness

A histogram of the numbness scores for the 394 knees showed that no numbness (numbness score = 4 points) was found in 170 (43.1%) knees and that some degree of numbness (numbness score ≤ 3 points) was found in the remaining 224 (56.9%) knees (Fig. 2). Since the distribution of numbness scores was quite skewed, with 43.1% having a numbness score of 4, the ICC (2,1) for the subset of patients who reported at least some numbness (score 0–3) was calculated and was found to be 0.68.

The results for each PROM were obtained from the last follow-up. The median numbness score in this cohort was 3 points (almost never aware of numbness around the surgical scar of the operated knee). For KSS-Kneeling, 0 points is the lowest score (cannot kneel) and 5 points is the highest score (can kneel without aggravation or numbness bother). The median score on the KSS-Kneeling was 2 points (kneel with severe discomfort). Therefore, kneeling after knee replacement in the entire cohort showed a high degree of discomfort (Table 4).

**Table 4:** Numerical data for each PROM, including numbness score and KSS-Kneeling

Scales of PROMs	Mean	Median	SD	Range
Numbness score: (0–4 points)	2.6	3	1.5	0–4
KSS				
Symptoms: (0–25 points)	19.7	21	5.8	0–25
Satisfaction: (0–40 points)	27.6	28	8.5	0–40
Expectation: (0–15 points)	9.9	9	2.8	0–15
Activities: (0–100 points)	66.3	69	20.1	0–100
Total: (0–180 points)	123.5	126	30.8	6–178
Kneeling: (0–5 points)	1.8	2	1.7	0–5
KOOS				
Symptom: (0–100 points)	82.4	85.7	14.3	21.4–100
Pain: (0–100 points)	82.5	86.1	16.7	16.7–100
ADL: (0–100 points)	79.6	83.8	16.5	21.6–100
Sports: (0–100 points)	43.4	45	28.9	0–100
QOL: (0–100 points)	60.7	62.5	24.6	0–100
FJS-12: (0–100 points)	45.3	42.7	26.0	0–100

*ADL* Activities of daily living, *FJS-12* Forgotten Joint Score-12, *KOOS* Knee Injury and Osteoarthritis Outcome Score, *KSS* New Knee Society Score, *PROM* Patient-reported outcome measure, *QOL* Quality of life, *SD* Standard deviation

The numbness score showed weak to (0.19–0.39) moderate (0.41–0.44) correlations with each PROM and its subscales (Table 5).

**Table 5:** Correlations between the numbness score and each PROM

PROMs	Correlation	95% CI	*p*-value
KSS			
Symptoms	0.44^b^	0.35–0.51	**< 0.0001**
Satisfaction	0.41^b^	0.32–0.49	**< 0.0001**
Expectation	0.19^a^	0.09–0.28	**< 0.0001**
Activities	0.29^a^	0.21–0.39	**< 0.0001**
Total	0.39^a^	0.30–0.47	**< 0.0001**
KOOS			
Symptom	0.42^b^	0.34–0.50	**< 0.0001**
Pain	0.44^b^	0.36–0.52	**< 0.0001**
ADL	0.36^a^	0.27–0.44	**< 0.0001**
Sports	0.23^a^	0.13–0.32	**< 0.0001**
QOL	0.38^a^	0.29–0.46	**< 0.0001**
FJS-12	0.42^b^	0.33–0.49	**< 0.0001**

*ADL* Activities of daily living, *CI* Confidence interval, *FJS-12* Forgotten Joint Score-12, *KOOS* Knee Injury and Osteoarthritis Outcome Score, *KSS* New Knee Society Score, *PROM* patient-reported outcome measure, *QOL* Quality of life; ^a^, Weak correlation; ^b^, Moderate correlation

Boldface means statistically significant

The univariable and multivariable regression analyses with the numbness score as the response variable suggested that the numbness score was better after midline incision than after anteromedial incision (Table 6).

**Table 6:** Results of univariable and multivariable regression analyses with numbness score as the response variable

Variables	Univariable			Multivariable		
	regression coefficient	95% CI	*P*-value	regression coefficient	95% CI	*P*-value
Age at surgery (years)	0.0049	− 0.01–0.02	0.58	0.001	− 0.02–0.02	0.91
Follow−up (months)	0.004	− 0.001–0.003	0.12			
BMI at time of surgery (kg/m^2^)	−0.04	−0.07 to − 0.04	**0.03**	−0.03	−0.07–0.004	0.08
Preoperative knee flexion angle (°)	0.004	−0.05–0.01	0.40			
Postoperative knee flexion angle (°)	0.009	−0.003–0.02	0.17			
Sex (male)	0.2	− 0.16–0.56	0.27	0.21	−0.18–0.59	0.29
Diagnosis			0.06			
ON [vs. OA]	0.61	0.07–1.16	**0.03**			
RA [vs. OA]	−0.21	−0.78–0.37	0.48			
Preoperative K − L grade			0.14			
K − L grade 2 [vs. Grade 1]	0.95	−1.91–3.81	0.51	1.38	−1.47–4.42	0.27
K − L grade 3 [vs. Grade 1]	1.61	−1.20–4.42	0.26	2.12	−0.69–4.93	0.14
K − L grade 4 [vs. Grade 1]	1.64	−1.15–4.44	0.25	2.32	−0.49–5.13	0.11
Surgical method [UKA]	0.3	−0.07–0.66	0.11	0.29	−0.19–0.78	0.23
Incision [Midline]	0.34	0.05–0.62	**0.02**	0.35	0.003–0.69	**0.04**
Arthrotomy			0.08			
Midvastus [vs. Medial parapatellar]	0.19	−0.23–0.60	0.38			
Subvastus [vs. Medial parapatellar]	−0.23	−0.58–0.12	0.20			
Trivector [vs. Medial parapatellar]	0.39	−0.41–1.20	0.34			

*CI* Confidence interval, *BMI* Body mass index, *K − L* Kellgren−Lawrence, *OA* Osteoarthritis, *ON* Osteonecrosis, *RA* Rheumatoid arthritis, *UKA* Unicondylar knee arthroplasty

Boldface means statistically significant

The univariable and multivariable regression analyses with KSS-Kneeling as the response variable suggested that male sex, better postoperative knee flexion, and better numbness score were factors associated with better kneeling ability (Table 7).

**Table 7:** Results of univariable and multivariable regression analyses with KSS − kneeling as the response variable

Variables	Univariable			Multivariable		
	Regression coefficient	95% CI	*P*-value	Regression coefficient	95% CI	*P*-value
Age at surgery (years)	−0.009	−0.03–0.01	0.40	−0.02	−0.04–0.02	0.50
Follow-up (months)	−0.008	−0.01 to − 0.002	**0.01**	−0.0008	−0.01–0.009	0.87
BMI at time of surgery (kg/m^2^)	−0.007	−0.05–0.03	0.71	−0.006	−0.05–0.03	0.75
Preoperative knee flexion angle (°)	0.002	−0.009–0.01	0.78			
Postoperative knee flexion angle (°)	0.02	0.008–0.04	**0.002**	0.01	0.0004–0.03	**0.04**
Sex [male]	0.92	0.51–1.33	**< 0.0001**	0.94	0.53–1.34	**< 0.0001**
Diagnosis			0.27			
ON [vs. OA]	0.21	−0.43–0.86	0.51			
RA [vs. OA]	0.52	−0.15–1.20	0.13			
Preoperative K–L grade			0.80			
K–L grade 2 [vs. Grade 1]	−1.47	−4.86–1.91	0.39			
K–L grade 3 [vs. Grade 1]	−1.19	−4.51–2.14	0.48			
K–L grade 4 [vs. Grade 1]	−1.21	−4.51–2.10	0.47			
Surgical method [UKA]	0.42	−0.008–0.85	0.05	0.38	−0.23–0.99	0.22
Incision [Midline]	−0.20	−0.53–0.14	0.25	−0.44	−0.96–0.07	0.09
Arthrotomy			0.48			
Midvastus [vs. Medial parapatellar]	0.26	−0.23–0.74	0.30			
Subvastus [vs. Medial parapatellar]	0.33	−0.08–0.74	0.11			
Trivector [vs. Medial parapatellar]	0.25	−0.70–1.20	0.61			
Numbness score	0.22	0.11–0.33	**0.0002**	0.19	0.07–0.30	**0.001**

*CI* Confidence interval, *BMI* Body mass index, *K − L* Kellgren–Lawrence, *OA* Osteoarthritis, *ON* Osteonecrosis, *RA* Rheumatoid arthritis, *UKA* Unicondylar knee arthroplasty

Boldface means statistically significant

## Discussion

The most important findings of this study were that greater numbness (lower numbness score) correlated with poor postoperative PROMs and greater numbness adversely affected kneeling. Therefore, our hypothesis was verified. The numbness score had moderate correlations with PROMs related to symptoms, joint awareness, and satisfaction. Multivariate regression analysis revealed that compared with midline incision, anteromedial incision was a risk factor for a worse (lower) numbness score. Better (higher) numbness score, male sex, and better knee flexion angle positively affected kneeling ability.

Several studies, such as the Oxford Knee Score (OKS), KSS, WOMAC, and KOOS [11, 13, 14], have reported that numbness did not correlate with PROMs. However, the present study demonstrated that our numbness score showed weak correlation with KSS-Expectation, KSS-Activities, KSS-Total, KOOS-Activities of Daily Living (ADL), KOOS-Sports, and KOOS-Quality of Life (QOL), and moderate correlations with KSS-Symptoms, KSS-Satisfaction, KOOS-Symptoms, KOOS-Pain, and the FJS-12. The numbness score may detect symptoms related to not only hyperesthesia but also to other sensory abnormalities because our numbness score method was more subjective than the numbness assessment methods used in previous reports [13, 31]. Therefore, the numbness score could detect numbness experienced by patients in a broad sense, including joint awareness, and was thus correlated with each PROM and its subscales.

In the current study, compared with midline incision, anteromedial incision was a risk factor for poor numbness score (univariable: regression coefficient = 0.34, 95% CI = 0.05–0.62, *p* = 0.02, multivariable: regression coefficient = 0.35, 95% CI = 0.003–0.69, *p* = 0.04). Several studies on the relationship between incision and numbness have been reported [26, 32, 33]. Similar to the results of our study, the results of Hassaballa et al. [33] showed that anteromedial incision caused a wider area of alteration in skin sensation than midline incision in a cohort of 38 TKAs and 40 UKAs with a minimum of 18 months of follow-up. Additionally, Laffose et al. [26] compared 31 TKAs performed via an anterolateral incision with 32 TKAs performed via a midline incision and found that an anterolateral incision resulted in a smaller area of numbness around the knee and that a smaller area of numbness correlated with better outcomes on the WOMAC and KOOS. A medially located skin incision could cause a wider area of paresthesia on the front of the knee because sensory nerve branches run across the front of the knee from the medial side [34]. In general, many knee arthroplasties are performed through a medial arthrotomy, which provides excellent surgical exposure in combination with midline or anteromedial incision [26], but anterolateral incision requires a longer incision and operation time [32], which is why there was no patient with an anterolateral incision in our cohort. A midline incision does not necessarily prevent numbness but appears to decrease postoperative numbness around the knee relative to that for anteromedial incision.

Our study results showed that kneeling ability was better for male sex than for female sex (univariable: regression coefficient = 0.92, 95% CI = 0.51–1.33, *p* < 0.0001; multivariable: regression coefficient = 0.94, 95% CI = 0.53–1.34, *p* < 0.0001). As in our study, Smith et al. [35] reported that kneeling was easier for men than for women. A meta-analysis of 29,993 TKA cases reported that women tended to complain more about persistent postoperative pain than men [36]. The results of that study support those of this study showing that women complained more of kneeling difficulties.

The current study revealed that better postoperative knee flexion was significantly associated with better kneeling (univariable: regression coefficient = 0.02, 95% CI = 0.008–0.04, *p* = 0.002, multivariable: regression coefficient = 0.01, 95% CI = 0.0004–0.003, *p* = 0.04). In a biomechanical study of TKA on cadaveric knees, Wilkens et al. [37] reported that patellofemoral contact pressure increased with knee flexion angles of 90°–120° in kneeling, but the pressure decreased for angles > 120° and decreased to the same degree as the non-flexion knee at 135°. Their study results appear to support the present study results showing that kneeling ability was better with greater knee flexion angle.

The univariable and multiple regression analyses indicated that lower numbness score (greater numbness) was a risk factor of kneeling difficulty (univariable: regression coefficient = 0.22, 95% CI = 0.11–0.33, *p* = 0.0002, multivariable: regression coefficient = 0.19, 95% CI = 0.07–0.30, *p* = 0.001). Several authors have reported correlations between numbness and kneeling ability [32, 33]. Tsukada et al. [32] reported that a larger area of numbness in the front of knees post-TKA negatively correlated with kneeling. Additionally, in a study by Hassaball et al. [33], patients post-TKA or UKA who could not kneel had significantly wider surface area of sensitivity and hypersensitivity in the front of their knees than those who could kneel. However, in a study that included 49 TKAs, Sharkey et al. [11] found that numbness did not correlate with the OKS, which includes items related to subjective symptoms, such as pain, and items related to complex movements, such as kneeling. In the present study with a larger cohort (394 knees) than in Sharkey’s study, subjective numbness around the surgical wound correlated with both subjective symptoms and kneeling, a result that differed from their report.

No report has shown that UKA provides better ability to kneel than TKA. When compared with TKA, the current study suggested that UKA had a positive effect on kneeling in univariate analysis, although the result was not significant (*p* = 0.054) and did not significantly affect kneeling in the multivariate analysis (*p* = 0.22). Similar to our study, Hassaballa et al. reported that UKA was not significantly advantageous over TKA in kneeling ability. Artz et al. [38] reported that approximately 35% of post-UKA and 45% of post-TKA patients could not kneel, but they did not state whether or not the difference was significant. Kneeling is affected by multiple factors and may be less affected by the difference between UKA and TKA.

Numbness is a minor complication, but it can correlate with symptoms, such as joint awareness and pain, and can affect functions, including kneeling. In our study, more than half (56.9%) of the patients experienced some degree of numbness around the surgical wound after knee arthroplasty. Based on this finding, surgeons can share with patients the prediction of possible postoperative symptoms by providing information on the relationship between numbness and clinical outcomes before surgery during the informed consent process. In addition, this finding may help surgeons choose surgical techniques since we found that an anteromedial incision may be a risk factor for postoperative numbness.

In our cohort, 514 knee arthroplasties (mean age, 72.9 years) were performed for about 12 years, which was a comparable rate of 40 women aged 70–74 years who underwent TKA per 10,000 per year in Denmark [39]. Based on the rates of knee arthroplasty and results of the power analysis described above, the sample size for this study was judged to be appropriate.

This study had several limitations. First, the numbness score did not objectively assess the location of the numbness. Previous studies have adopted objective assessments for numbness [13, 31]. In contrast, this study adopted a subjective assessment using a numbness score that asked about numbness “around the surgical wound”. Therefore, the numbness score may not have reflected the exact nature and location of the numbness complained of by the patients. However, the reliability of the numbness score was confirmed by calculating the ICC (see the section on “statistical analysis”), so the numbness score was associated with the degree of patient-reported numbness in our study. Second, a retrospective study design was used, and the mean follow-up duration of 28.0 months was relatively short. It has been reported that complaints of numbness were more common in the first 3 years after TKA than from 6 years onward after TKA [40], suggesting that numbness would decrease as the duration increases. Therefore, our mean follow-up may have occurred when numbness was frequent. Third, patients who underwent bilateral surgeries, which can be a confounder, were included, but the patients were asked to report PROMs for each knee independently. As a reference, correlation and regression analyses were performed on the data based on 311 knees in 311 patients, excluding patients who had undergone surgery on both knees. The results were comparable to those obtained from the 394 knees (Additional file 1). Fourth, the cohort underwent knee replacement surgery over a relatively long period of 12 years, from May 2007 to January 2019. Over these 12 years, there have been advances in perioperative management with the development of the enhanced recovery after surgery (ERAS) program and surgical techniques [6, 41]. There was no significant change in the surgical technique of TKA in our cohort over 12 years, as TKA was consistently performed using conventional instruments to achieve mechanical alignment. Although ERAS has been reported to reduce early postoperative complications and shorten hospital stays [41], the effect of ERAS on PROMs at a mean of 28 months after surgery is unknown in this cohort. Fifth, although psychiatric disorders such as depression and anxiety have been reported to affect outcomes after knee arthroplasty [41, 42], the present study did not exclude patients with psychiatric disorders, which may have affected the PROMs. However, Buller et al. reported that 4.1 and 1.7% of 8,379,490 patients who underwent TKA or total hip arthroplasty had depressive disorders and anxiety disorders, respectively, [42] and it is expected that the general cohort after knee arthroplasty would include patients with some form of psychiatric disorders. Therefore, patients with psychiatric disorders in our cohort were not excluded.

## Conclusions

Patient-reported numbness around the surgical wound after knee replacement correlated with postoperative PROMs and affected kneeling. The numbness score mainly correlated with symptoms, patient satisfaction, and joint awareness. Compared with midline incision, anteromedial incision was found to be a risk factor of lower numbness score (greater numbness). Higher numbness score (less numbness), male sex, and better knee flexion angle were positively associated with better kneeling ability.

## Supplementary Information

**Additional file 1.**

## Data Availability

The datasets used and/or analyzed during the study are available from the corresponding author upon reasonable request.

## Abbreviations

ADL: Activity of daily living
BMI: Body mass index
CI: Confidence interval
FJS-12: Forgotten Joint Score-12
ERAS: Enhanced recovery from surgery
HTO: High tibial osteotomy
ICC: Intraclass correlation
K-L: Kellgren–Lawrence
KOOS: Knee Injury and Osteoarthritis Outcome Score
KSS: New Knee Society Score
MCL: Medial collateral ligament
OA: Osteoarthritis
OKS: Oxford knee score
ON: Osteonecrosis
PROM: Patient-reported outcome measure
QOL: Quality of life
RA: Rheumatoid arthritis
SD: Standard deviation
TKA: Total knee arthroplasty
UKA: Unicondylar knee arthroplasty
WOMAC: Western Ontario and McMaster Universities Osteoarthritis Index
