# Supplementary material for: Correlation of patient-reported numbness around surgical scars with patient-reported outcome measures and joint awareness after knee replacement: a cohort study
Source: BMC Musculoskelet Disord. 2022 Jan 3;23:14. doi: 10.1186/s12891-021-04971-6 (PMC8725250; doi:10.1186/s12891-021-04971-6)
Supplement: Supplementary file 1 — Additional file 1. [file 12891_2021_4971_MOESM1_ESM.docx]

Table 5a

Correlations between the numbness score and PROMs for 311 knees in 311 patients

| PROMs | Correlation | 95% CI | *p*-value |
| --- | --- | --- | --- |
| KSS |  |  |  |
| Symptoms | 0.43** | 0.34–0.52 | **<0.0001** |
| Satisfaction | 0.42** | 0.33–0.51 | **<0.0001** |
| Expectation | 0.20* | 0.09–0.30 | **<0.0001** |
| Activities | 0.28* | 0.18–0.38 | **<0.0001** |
| Total | 0.38* | 0.29–0.47 | **<0.0001** |
| KOOS |  |  |  |
| Symptom | 0.42** | 0.32–0.50 | **<0.0001** |
| Pain | 0.44** | 0.35–0.52 | **<0.0001** |
| ADL | 0.35* | 0.25–0.44 | **<0.0001** |
| Sports | 0.22* | 0.11–0.32 | **<0.0001** |
| QOL | 0.38* | 0.28–0.47 | **<0.0001** |
| FJS-12 | 0.42** | 0.33–0.51 | **<0.0001** |

ADL, activities of daily living; CI, confidence interval; FJS-12, Forgotten Joint Score-12; KOOS, Knee Injury and Osteoarthritis Outcome Score; KSS, New Knee Society Score; PROM, patient-reported outcome measure; QOL, quality of life; *, Weak correlation; **, Moderate correlation.

Boldface means statistically significant.

Table 6aResults of univariable and multivariable regression analyses with numbness score as the response variable for 311 knees in 311 patients

| Variables | Univariable | | |  | Multivariable | | |
| --- | --- | --- | --- | --- | --- | --- | --- |
|  | regression coefficient | 95% CI | *p*-value |  | regression coefficient | 95% CI | *p*-value |
| Age at surgery (years) | 0.0046 | −0.019–0.020 | 0.96 |  | -0.003 | −0.03–0.02 | 0.79 |
| Follow-up (months) | 0.003 | −0.003–0.009 | 0.32 |  |  |  |  |
| BMI at time of surgery (kg/m^2^) | −0.04 | −0.07–0.004 | 0.08 |  | −0.04 | −0.08–0.007 | 0.10 |
| Preoperative knee flexion angle (°) | 0.004 | −0.006–0.015 | 0.37 |  |  |  |  |
| Postoperative knee flexion angle (°) | 0.010 | −0.003–0.02 | 0.13 |  |  |  |  |
| Sex (% male) | 0.21 | −0.17–0.59 | 0.28 |  | 0.20 | −0.22–0.61 | 0.35 |
| Diagnosis |  |  | 0.11 |  |  |  |  |
| ON [vs. OA] | 0.56 | −0.01–1.14 | 0.06 |  |  |  |  |
| RA [vs. OA] | −0.24 | −0.86–0.38 | 0.45 |  |  |  |  |
| Preoperative K−L grade |  |  | 0.24 |  |  |  |  |
| K−L grade 2  [vs. Grade 1] | 1.06 | −1.83–3.83 | 0.49 |  | 1.50 | −1.40–4.40 | 0.39 |
| K−L grade 3  [vs. Grade 1] | 1.73 | −1.12–4.44 | 0.23 |  | 2.26 | −0.60–5.11 | 0.12 |
| K−L grade 4  [vs. Grade 1] | 1.60 | −1.23–4.44 | 0.27 |  | 2.30 | −0.56–5.16 | 0.11 |
| Surgical method [UKA] | 0.40 | 0.007–0.78 | **0.046** |  | 0.34 | −0.19–0.86 | 0.21 |
| Incision [Midline] | 0.36 | 0.07–0.69 | **0.02** |  | 0.34 | 0.003–0.66 | **0.04** |
| Arthrotomy |  |  | 0.06 |  |  |  |  |
| Midvastus [vs. Medial parapatellar] | 0.29 | −0.16–0.74 | 0.21 |  |  |  |  |
| Subvastus [vs. Medial parapatellar] | −0.23 | −0.62–0.17 | 0.26 |  |  |  |  |
| Trivector [vs. Medial parapatellar] | 0.24 | −0.76–1.24 | 0.64 |  |  |  |  |

CI, confidence interval; BMI, body mass index; K−L, Kellgren−Lawrence; OA, osteoarthritis; ON, osteonecrosis; RA, rheumatoid arthritis; UKA, unicondylar knee arthroplasty.

Boldface means statistically significant.

Table 7a

Results of univariable and multivariable regression analyses with KSS−Kneeling as the response variable for 311 knees in 311 patients

| Variables | Univariable | | |  | Multivariable | | |
| --- | --- | --- | --- | --- | --- | --- | --- |
|  | Regression coefficient | 95% CI | *p*-value |  | Regression coefficient | 95% CI | *p*-value |
| Age at surgery (years) | −0.01 | −0.04–0.009 | 0.23 |  | −0.02 | −0.04–0.02 | 0.32 |
| Follow-up (months) | −0.009 | −0.02 to −0.002 | **0.01** |  | −0.0007 | −0.01–0.010 | 0.90 |
| BMI at time of surgery (kg/m^2^) | −0.01 | −0.06–0.03 | 0.57 |  | -0.016 | −0.06–0.03 | 0.48 |
| Preoperative knee flexion angle (°) | 0.0003 | −0.01–0.01 | 0.95 |  |  |  |  |
| Postoperative knee flexion angle (°) | 0.02 | 0.008–0.04 | **0.002** |  | 0.015 | 0.0004–0.03 | **0.048** |
| Sex (% male) | 0.86 | 0.43–1.29 | **<0.0001** |  | 0.88 | 0.45–1.30 | **<0.0001** |
| Diagnosis |  |  | 0.35 |  |  |  |  |
| ON [vs. OA] | 0.06 | −0.60–0.73 | 0.85 |  |  |  |  |
| RA [vs. OA] | 0.53 | −0.19–1.24 | 0.15 |  |  |  |  |
| Preoperative K−L grade |  |  | 0.74 |  |  |  |  |
| K−L grade 2 [vs. Grade 1] | −1.39 | −4.76–1.98 | 0.42 |  |  |  |  |
| K−L grade 3 [vs. Grade 1] | −1.00 | −4.31–2.31 | 0.55 |  |  |  |  |
| K−L grade 4 [vs. Grade 1] | −1.16 | −4.44–2.13 | 0.49 |  |  |  |  |
| Surgical method [UKA] | 0.47 | 0.02–0.91 | **0.04** |  | 0.50 | −0.15–1.15 | 0.13 |
| Incision [Midline] | −0.20 | −0.56–0.17 | 0.29 |  | −0.50 | −1.07–0.06 | 0.08 |
| Arthrotomy |  |  | 0.43 |  |  |  |  |
| Midvastus [vs. Medial parapatellar] | 0.31 | −0.21–0.84 | 0.24 |  |  |  |  |
| Subvastus [vs. Medial parapatellar] | 0.34 | −0.11–0.80 | 0.14 |  |  |  |  |
| Trivector [vs. Medial parapatellar] | -0.11 | −1.27–1.05 | 0.86 |  |  |  |  |
| Numbness score | 0.17 | 0.05–0.29 | **0.007** |  | 0.13 | 0.009–0.26 | **0.03** |

CI, confidence interval; BMI, body mass index; K−L, Kellgren–Lawrence; OA, osteoarthritis; ON, osteonecrosis; RA, rheumatoid arthritis; UKA, unicondylar knee arthroplasty.

Boldface means statistically significant.
